# Quantification of emotional bias by an Emotional-Gain Model

**DOI:** 10.1186/1471-2202-12-S1-P326

**Published:** 2011-07-18

**Authors:** David Nicoladie Tam

**Affiliations:** 1 Department of Biological Sciences, University of North Texas, Denton, TX 76203, USA

## 

We have developed a computational model that quantifies emotion objectively based on neurobiological mechanisms that increases the chance of survival in the real world. This model is based on the EMOTION-I and EMOTION-II models [1,2] with the extension of proportionality hypothesis added to the desirable gain signal to these models. The emotion derived from this model identified emotion as a feedback mechanism to detect self-derived error of the internal brain model that represents the real world. The survivability of animal depends on this autonomous ability to self-discover errors within its internal model of the world, such that correction can be made to obtain a more accurate model of the external world. The self-discovered error is derived from comparing the difference between the actual reality and the internal model’s prediction. This represents the difference between the objective and subjective realities. The discrepancy signal serves as the feedback signal for error-detection. This discrepancy signal becomes a quantitative measure of the difference between what one wants and gets. If the difference signal represents the desirable goal, that becomes the desirable gain signal for the error-feedback. This desirable gain is hypothesized to be proportional to the intensity of happy emotion in this Emotion-Gain model. Conversely, the intensity of unhappy emotion is inversely proportional to the desirable gain.

This Emotional-Gain Model can represent the emotional intensity and the desirable gain graphically. The emotional bias is represented quantitatively by the shifting of the emotional curve in this Emotional-Gain space, either up-or-down, left-or-right or change in the slope of the curve. Confirmation of this proportional hypothesis is provided by using the Ultimatum Game (UG) with human subjects. The results show that happy emotion is proportional to the desirable gain, and unhappy emotion is inversely proportional to the desirable gain [3]. The emotional biases are also quantified the shifting of the emotional curve relative to the acceptance and rejection decision in the UG experiment.
